# A Complex Distribution of Elongation Family GTPases EF1A and EFL in Basal Alveolate Lineages

**DOI:** 10.1093/gbe/evu186

**Published:** 2014-08-31

**Authors:** Kirill V. Mikhailov, Jan Janouškovec, Denis V. Tikhonenkov, Gulnara S. Mirzaeva, Andrei Yu. Diakin, Timur G. Simdyanov, Alexander P. Mylnikov, Patrick J. Keeling, Vladimir V. Aleoshin

**Affiliations:** ^1^Belozersky Institute for Physico-Chemical Biology, Lomonosov Moscow State University, Russian Federation; ^2^Institute for Information Transmission Problems, Russian Academy of Sciences, Moscow, Russian Federation; ^3^Botany Department, University of British Columbia, Vancouver, British Columbia, Canada; ^4^Institute for Biology of Inland Waters, Russian Academy of Sciences, Borok, Yaroslavl Province, Russian Federation; ^5^Institute of Gene Pool of Plants and Animals, Uzbek Academy of Sciences, Tashkent, Republic of Uzbekistan; ^6^Botany and Zoology, Faculty of Science, Masaryk University, Brno, Czech Republic; ^7^Faculty of Biology, Lomonosov Moscow State University, Russian Federation; ^8^National Research Institute of Physiology, Biochemistry, and Nutrition of Farm Animals, Russian Academy of Agricultural Sciences, Borovsk, Kaluga Region, Russian Federation

**Keywords:** Elongation Factors, Alveolata, EF1A, EFL, Colpodellids, Chromerids, Colponemids

## Abstract

Translation elongation factor-1 alpha (EF1A) and the related GTPase EF-like (EFL) are two proteins with a complex mutually exclusive distribution across the tree of eukaryotes. Recent surveys revealed that the distribution of the two GTPases in even closely related taxa is frequently at odds with their phylogenetic relationships. Here, we investigate the distribution of EF1A and EFL in the alveolate supergroup. Alveolates comprise three major lineages: ciliates and apicomplexans encode EF1A, whereas dinoflagellates encode EFL. We searched transcriptome databases for seven early-diverging alveolate taxa that do not belong to any of these groups: colpodellids, chromerids, and colponemids. Current data suggest all seven are expected to encode EF1A, but we find three genera encode EFL: *Colpodella*, *Voromonas*, and the photosynthetic *Chromera*. Comparing this distribution with the phylogeny of alveolates suggests that EF1A and EFL evolution in alveolates cannot be explained by a simple horizontal gene transfer event or lineage sorting.

## Introduction

Translation elongation factor-1 a (EF1A) is a key component of protein synthesis in eukaryotes, recruiting charged tRNAs to the ribosome (and is homologous to archaeal EF1A and bacterial EF-Tu, which fulfill the same role). Despite this essential function, however, widespread genomic analysis of diverse eukaryotes has shown a significant number of lineages lack EF1A, and in all such cases encode a second related subfamily of GTPase called EF-like (EFL) (Gile et al. 2006; Ruiz-Trillo et al. 2006; Noble et al. 2007; Gile and Keeling 2008; Gile, Faktorová, et al. 2009; Sakaguchi et al. 2009; Kamikawa et al. 2011). EFL is assumed to fulfill the essential function of EF1A because of their mutually exclusive distribution, and because EFL retains the main binding sites of functional significance (Keeling and Inagaki 2004).

The distribution of EF1A and EFL has been difficult to explain by any simple model since it was first discovered. Neither protein is restricted to a group of closely related lineages, and instead both proteins are widely scattered among different subgroups in the tree of eukaryotes. This complex distribution led initial reports to question whether the current pattern was due to ancient paralogy and lineage sorting, more recent horizontal gene transfer (HGT), or a combination of both. HGT has become the favored explanation (Keeling and Inagaki 2004; Kamikawa et al. 2008; Sakaguchi et al. 2009), although in at least one case the pattern is more consistent with lineage sorting (Gile, Faktorová, et al. 2009). Distinguishing between the two is difficult because the great diversity of taxa involved and ancient time scales of the events, which both contribute to insufficient resolution in the phylogenies to unequivocally document cases of HGT or lineage sorting (Cocquyt et al. 2009; Kamikawa et al. 2010). EFL has now been found in lineages from all major eukaryotic supergroups, and its overall distribution has become even more complex (Ruiz-Trillo et al. 2006; Cocquyt et al. 2009; Gile, Faktorová, et al. 2009; Sakaguchi et al. 2009; Cavalier-Smith and Chao 2012; Henk and Fisher 2012; Ishitani et al. 2012; Kamikawa et al. 2013). But more interestingly, deeper analyses into some EFL-containing lineages have shown that the distribution patterns between closely related lineages may also be complex; this is particularly well documented in green algae and euglenozoa, where very unusual distribution patterns conflict with known phylogenetic relationships (Gile, Faktorová, et al. 2009; Gile, Novis, et al. 2009).

In the alveolates, a major eukaryotic supergroup comprising the well-studied lineages ciliates, dinoflagellates, and apicomplexans, only the dinoflagellates and their close relative *Perkinsus* have EFL, whereas all other alveolates have EF1A (Gile et al. 2006). Previous work on EFL-containing taxa rejected the monophyletic origin of EFL gene in *Perkinsus* and dinoflagellates, suggesting independent transfers of EFL gene in closely related groups (Gile et al. 2006). Here, we show that sampling a number of early-diverging alveolate lineages requires the addition of lineage sorting events to this pattern.

## Early-Diverging Alveolates Have Either EF1A or EFL

In addition to the three major lineages of alveolates, molecular and morphological data have both shown that photosynthetic chromerids (*Chromera* and *Vitrella*) and predatory colpodellids (*Colpodella*, *Voromonas*, and *Alphamonas*) are basal relatives of the apicomplexans (Kuvardina et al. 2002; Leander et al. 2003; Moore et al. 2008; Janouškovec et al. 2010; Obornik et al. 2012; Gile and Slamovits 2014), and the enigmatic colponemid predators also branch deeply within the alveolates (Janouškovec et al. 2013; Tikhonenkov et al. 2014). We searched transcriptome databases from representatives of all six genera (and in the case of *Acavomonas peruviana* targeted polymerase chain reaction [PCR]) for homologues of EF1A and EFL. Based on the organismal phylogeny and current distribution of the proteins, the expectation would be that all these taxa should encode EF1A and not EFL, but EF1A was only found in colponemids, *Vitrella*, and *Alphamonas*. Surprisingly, EFL was found in *Colpodella*, *Voromonas*, and *Chromera*. In no case were both genes found in the same taxon suggesting that only one type is expressed or present. No additional EF1A or EFL paralogs were detected in the transcriptome assemblies or the previously prepared DNA library or a small-scale genome sequence survey of *A. peruviana* (Janouškovec et al. 2013). Incidentally, we also found two different elongation factors in bodonids that were used as prey for predatory colpodellids, with *Parabodo caudatus* encoding EF1A and *Procryptobia sorokini* encoding EFL, further supporting previous work on the distribution of the two proteins in kinetoplastids (Gile, Faktorová, et al. 2009).

**Fig. 1.—:**
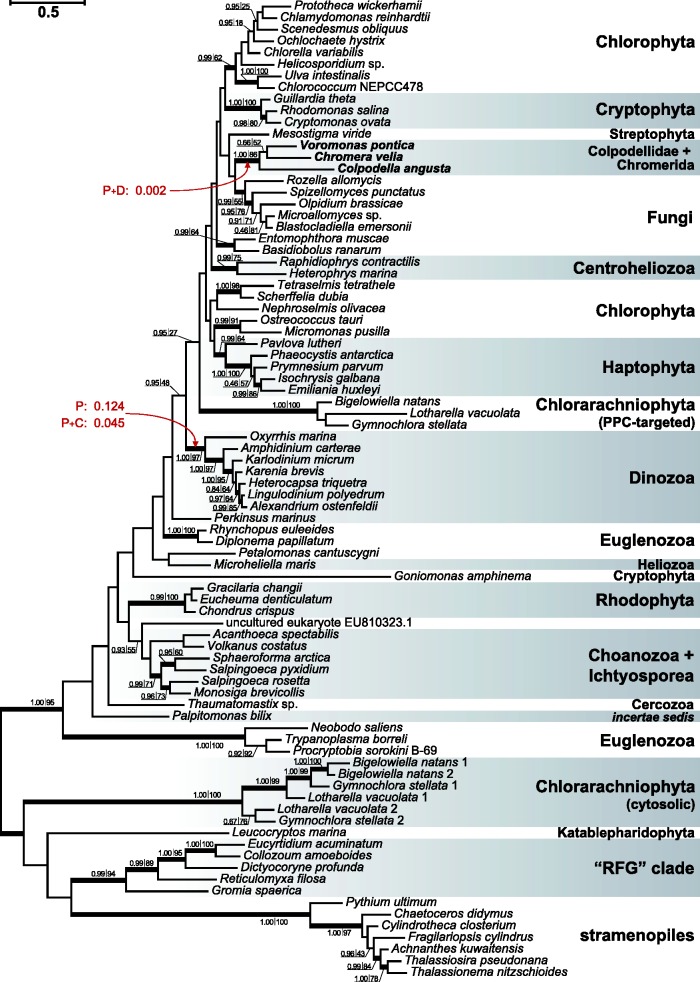
Phylogeny of EFL. The tree was reconstructed using Bayesian inference (PhyloBayes) under CAT profile mixture model with four discrete gamma categories and the exchange rates fixed by the LG model (maxdiff = 0.127; loglik effsize = 188). Node support values are given for two types of tree inference methods—Bayesian posterior probability (left) and maximum–likelihood (ML) bootstrap support value (right); bootstrap support was generated on the basis of 1,000 replicates using RAxML and LG+G+F model. Support values for nodes with Bayesian posterior probabilities <0.95 and ML bootstrap support <50% are omitted. Nodes with Bayesian posterior probabilities ≥0.95 and ML bootstrap support ≥50% are given with thick lines. The “RFG” clade stands for Radiolaria, Foraminifera, and *Gromia*—a tentative group introduced in Ishitani et al. (2012). PPC, periplastid compartment.

**Fig. 2.—:**
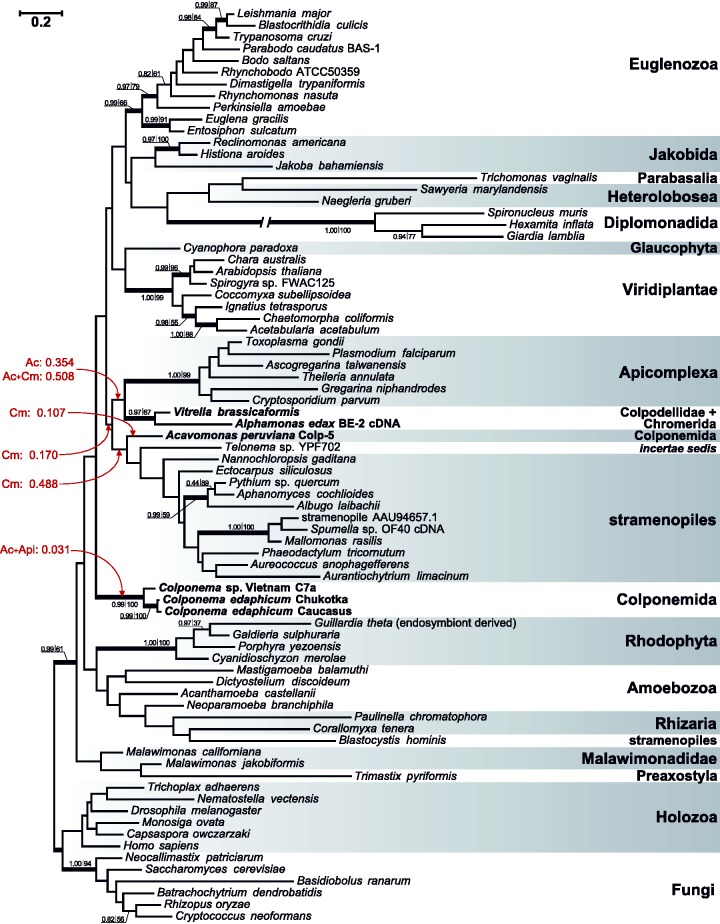
Phylogeny of EF1A. The tree was reconstructed using Bayesian inference (PhyloBayes) under CAT profile mixture model with four discrete gamma categories and the exchange rates fixed by the LG model (maxdiff = 0.244; loglik effsize = 113). Node support values are given for two types of tree inference methods—Bayesian posterior probability (left) and maximum-likelihood (ML) bootstrap support value (right); bootstrap support was generated on the basis of 1,000 replicates using RAxML and LG+G+I model. Support values for nodes with Bayesian posterior probabilities <0.95 and ML bootstrap support <50% are omitted. Nodes with Bayesian posterior probabilities ≥0.95 and ML bootstrap support ≥50% are given with thick lines. The branch leading to diplomonads, marked with a hatch, is artificially shortened.

Phylogenetic analysis places the EFL genes from the early-diverging alveolate lineages in a relatively well-supported clade (fig. 1), indicating a single common origin in these taxa. The relationship of these sequences to other alveolate EFLs from dinoflagellates and their close relative *Perkinsus* was not adequately supported, and they were found to branch very distantly in the tree: the coplodellid/chromerid branch fell much closer to green algae, cryptomonads and fungi, than they did to the dinoflagellates and *Perkinsus*. Using the EFL data, the monophyly of coplodellids/chromerids plus dinoflagellates/*Perkinsus* is rejected by the approximately unbiased (Shimodaira 2002), one sided Kishino–Hasegawa (Kishino and Hasegawa 1989), and Expected Likelihood Weight (Strimmer and Rambaut 2002) tests, but fails to be rejected by the two-sided Kishino–Hasegawa (Kishino and Hasegawa 1989) and Shimodaira–Hasegawa (Shimodaira and Hasegawa 1999) tests. Unlike previous analysis (Gile et al. 2006), the monophyly of *Perkinsus* and dinoflagellates was not rejected by the approximately unbiased and other tests with the taxon sampling used. In EF1A phylogenies, the ciliates have historically been shown to have a confounding covarion effect (Moreira et al. 1999), and so we analyzed this gene with and without ciliates included. In neither case was the alveolates recovered or were many of the relationships between the alveolate subgroups supported, but the *Vitrella* and *Alphamonas* sequences consistently branched at the base of the apicomplexans as one would expect (fig. 2 and supplementary fig. S1, Supplementary Material online). In both analyses, with and without ciliates, colponemid EF1A sequences formed two independent lineages, with *A. peruviana* having a loose association with the branch uniting stramenopiles and *Telonema*, whereas the other colponemid sequences branched elsewhere in the tree with no support (fig. 2 and supplementary fig. S1, Supplementary Material online). However, the monophyly of alveolates is not rejected with the EF1A data by the approximately unbiased and the more liberal tests. Resolution of EFs trees does not increase when the most variable positions are excluded from the alignments (supplementary figs. S2 and S3, Supplementary Material online). Overall, there is no evidence that any unusual evolutionary event such as HGT has affected alveolate EF1A, and the phylogeny of EFL can only be used to make a strong case that the colpodellid/chromerid EFLs arose in common, but whether they arose separately from dinoflagellate and *Perkinsus* EFLs cannot be concluded with strong support (at face value, the trees suggest separate origins).

## Alveolates EF1A and EFL Have a Complex Evolutionary History

**Fig. 3.—:**
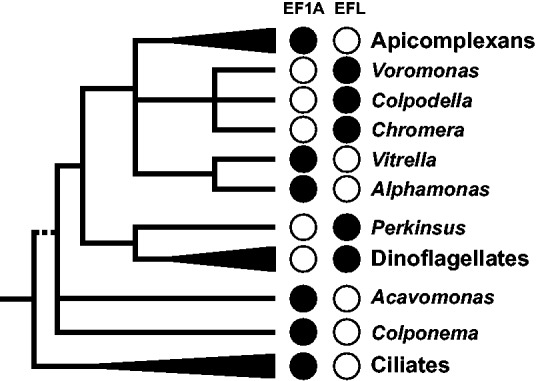
Schematic diagram of prospective relationships between the three main alveolate lineages and the early-diverging colponemids, perkinsids, colpodellids, and chromerids. The relationships are based on the rDNA phylogeny (supplementary fig. S4, Supplementary Material online and Gile and Slamovits 2014): Polytomies are unknown, and dotted lines less certain. The presence (filled circle) or absence (open circle) of EF1A and EFL is indicated for each branch.

Plotting the presence and absences of EF1A and EFL on alveolate phylogeny reveals no easy explanation for the current distribution of the translation elongation GTPases (fig. 3). If all alveolate EFLs originated only by HGT, there must have been two (colpodellids and all dinoflagellates), or perhaps even three (colpodellids, *Perkinsus*, and core dinoflagellates) individual events. If the distribution is only due to ancient paralogy and lineage sorting, then both genes must have coexisted for a prolonged period in the lineage leading to apicomplexans, leading to three independent sorting events at a minimum. The reality may lay between these two extremes, with a mix of HGT events followed by a period of redundancy and lineage sorting. This makes sense from a functional standpoint as well, as it seems unrealistic to expect an incoming transferred gene to simply replace its existing analog in an instant, so some period of more or less gradual change of function is not unreasonable, and during this period the functional assignment could likely go either way. The deepest alveolate lineages (colponemids, ciliates) seem to contain only EF1A gene but this circumstance does not lead to a definite conclusion, as both EF1A and EFL genes are present in the outgroup (at stramenopiles and Rhizaria). So the ultimate origin of EFL in alveolates may be HGT, but lineage sorting could still play a role in the current distribution if, for example, the ancestor of apicomplexan and dinoflagellate lineages acquired a copy of EFL by HGT, and the descendent lineages retained one or the other resulting in the pattern seen here. This complexity suggests ancestors of organisms with EF1A (e.g., apicomplexans) may have once also encoded EFL, and underscores the importance of sampling a broad taxonomic diversity when reconstructing such events. The early-diverging alveolate lineages are still poorly studied compared with model organisms from the three main lineages, but without more data from these organisms our understanding of the evolution of the main lineages will remain incomplete.

## Materials and Methods

Predatory flagellates *Colpodella angusta* isolate Spi-2 and *Colponema vietnamica* isolate Colp-7 a were cultured with free-living bodonid prey *Parabodo caudatus* strain BAS-1; *Voromonas pontica* isolate G-3 and *Alphamonas edax* isolate BE-2 were cultured with bodonid *P. sorokini* strain B-69 and heterotrophic chrysophyte *Spumella* sp. isolate OF-40, respectively. EFs from the prey and predator organisms were identified in the isolated prey and mixed transcriptomes generated using SMARTer Pico PCR cDNA Synthesis Kit (Clontech) and Illumina HiSeq sequencing, and assembled in Inchworm (Trinity v. r2012-06-08) using default parameters, according to the pipeline (Keeling et al. 2014). Between 14 and 48 million 100-bp paired-end raw reads were obtained per sample. The number of contigs assembled for each species ranged from 4 x 10^4^ to 1.7 x 10^5^ (of which a minimum was 40,668 contigs in *V. pontica* assembly). Up to 15% of contigs were discarded during filtering for prey contamination and even more are bacterial contaminants that could not be filtered out due to the lack of reference genomes. Predatory flagellates *A. peruviana* isolate Colp-5 and two isolates of *Colponema edaphicum* (Chukotka and Caucasus) were cultured with *P. sorokini* strain B-69 and *Spumella* sp. isolate OF-40, respectively. Alveolate EF1A was generated by PCR using Encyclo PCR kit (Evrogen) and a pair of degenerate primers (5′-GTTYAARTAYGCNTGGGTNYTNGA-3′, 5′-ATRTGVGMIGTRTGRCARTC-3′), and sequenced directly on Applied Biosystems 3730 DNA Analyzer. No EFL sequences of *A. peruviana* or *C. edaphicum* were detected by PCR or found in their transcriptomes. Sequences obtained in this study were deposited in GenBank with accession numbers KF997847–KF997856. EFs genes from *Chromera velia* and *Vitrella brassicaformis* were identified in transcriptomes generated through the Marine Microbial Eukaryote Transcriptome Sequencing Project (Gordon and Betty Moore Foundation).

New sequences were translated and aligned with a taxonomically broad sample of EF1A and EFL sequences collected from GenBank (nr, est, wgs), Joint Genome Institute, Broad Institute, and TBestDB databases. Elongation factor sequences of *Nannochloropsis gaditana*, *Pythium ultimum*, and *Galdieria sulphuraria* were extracted in their respective genome project databases (supplementary table S1, Supplementary Material online). The alignments of EF1A and EFL amino acid sequences were prepared separately using MUSCLE alignment program (Edgar 2004) and manually refined using BioEdit (Hall 1999). After the exclusion of ambiguously aligned positions, the EF1A data set contained 81 sequences and 419 positions, and the EFL data set contained 84 sequences and 452 positions. Tree search for both data sets was performed using the Bayesian method implemented by PhyloBayes 3.3 (Lartillot et al. 2009). Tree reconstruction for both data sets used the CAT profile mixture model with four discrete gamma categories and the exchange rates fixed by the LG model. For each data set, four independent chains were run for 50,000 cycles sampling trees every 100 cycles after discarding the first 10,000 cycles as burn-in. The maximum discrepancy (maxdiff parameter) had values less than 0.3, and the effective sizes (for loglik parameter) ranged from 58 to 188. For the specific parameter values related to individual trees, see the figure captions. The sampled trees were used to generate the majority rule consensus tree with Bayesian posterior probabilities. Bootstrap support values for the consensus tree reconstructed by PhyloBayes were generated using RAxML 7.2.6 (Stamatakis 2006) on the basis of 1,000 replicates under the LG+G+I model for the EF1A data set and LG+G+F model for the EFL data set. The models for each data set were chosen as best-fit by ModelGenerator 0.85 (Keane et al. 2006). The alternative topologies were tested using the CONSEL program (Shimodaira and Hasegawa 2001). The topologies were visualized using TreeView (Page 1996), and site-wise log-likelihood values were computed with TREE-PUZZLE program under the LG+G model (Schmidt 2009).

## Supplementary Material

Supplementary tables S1 and figures S1–S4 are available at *Genome Biology and Evolution* online (http://www.gbe.oxfordjournals.org/).

Supplementary Data
