# Supplementary material for: A Complex Distribution of Elongation Family GTPases EF1A and EFL in Basal Alveolate Lineages
Source: Genome Biol Evol. 2014 Aug 31;6(9):2361–7. doi: 10.1093/gbe/evu186 (PMC4217694; doi:10.1093/gbe/evu186)
Supplement: Supplementary Data [file supp_6_9_2361__index.html]

A complex distribution of elongation family GTPases EF1A and EFL in basal alveolate lineages — A Complex Distribution of Elongation Family GTPases EF1A and EFL in Basal Alveolate Lineages — Supplementary Data 

# A Complex Distribution of Elongation Family GTPases EF1A and EFL in Basal Alveolate Lineages

## Supplementary Data

files

**Files in this Data Supplement:**

- Supplementary Data - pdf file
