# Supplementary material for: A Complex Distribution of Elongation Family GTPases EF1A and EFL in Basal Alveolate Lineages
Source: Genome Biol Evol. 2014 Aug 31;6(9):2361–7. doi: 10.1093/gbe/evu186 (PMC4217694; doi:10.1093/gbe/evu186)
Supplement: Supplementary Data [file supp_evu186_Mikhailov_et_Supplementary_ver2.pdf]

Supplementary Table 1. List of databases, sequence identifiers and accessions used in the work.

Supplementary Figure 1. Phylogeny of EF1A with ciliates. The tree was reconstructed using Bayesian inference (PhyloBayes) under CAT profile mixture model with 4 discrete gamma categories and the exchange rates fixed by the LG model (maxdiff=0.217; loglik effsize=58). Node support values are given for two types of tree inference methods – Bayesian posterior probability (left) and maximum likelihood bootstrap support value (right); bootstrap support was generated on the basis of 1000 replicates using RAxML and LG+G+I model. Support values for nodes with Bayesian posterior probabilities <0.95 and ML bootrstrap support <50% are omitted. Nodes with Bayesian posterior probabilities ≥0.95 and ML bootrstrap support ≥50% are given with thick lines. The branch leading to diplomonads, marked with a hatch, is artificially shortened.

Supplementary Figure 2. Bayesian tree of EFL (PhyloBayes under CAT profile mixture model with 4 discrete gamma categories and the exchange rates fixed by the LG model) calculated after excluding the most variable 18.6% positions from the alignment.

Supplementary Figure 3. Bayesian tree of EF1A (PhyloBayes under CAT profile mixture model with 4 discrete gamma categories and the exchange rates fixed by the LG model) calculated after excluding the most variable 16.2% positions from the alignment.

Supplementary Figure 3. Bayesian tree of the alveolates SSU rDNA. Alternative positions of Colpodellida-1/Chomerida clade were tested with the approximately unbiased test, and the calculated *p*-values are indicated.

EF1A		
Name	Database	Accession
Acanthamoeba castellanii	TBestDB	ACL00002409
Acetabularia acetabulum	GenBank	EF551321.1
Albugo laibachii	GenBank	CCA22726.1
Aphanomyces cochlioides	GenBank	ABN54676.1
Arabidopsis thaliana	GenBank	AAL57653.1
Ascogregarina taiwanensis	GenBank	ABJQ01004964.1
Aurantiochytrium limacinum	DOE Joint Genome Institute	jgi|Aurli1|105065|estExt_Genewise1Plus.C_7_t10466
Aureococcus anophagefferens	DOE Joint Genome Institute	jgi|Auran1|36932
Basidiobolus ranarum	GenBank	DQ282610.1
Batrachochytrium dendrobatidis	DOE Joint Genome Institute	jgi|Batde5|19314|estExt_Genewise1Plus.C_40375
Blastocrithidia culicis	GenBank	ACO50122.1
Blastocystis hominis	GenBank	AAC36746.2
Blepharisma japonicum	GenBank	AAD03251.1
Bodo saltans	GenBank	ACO50123.1
Capsaspora owczarzaki	Broad Institute, Origins of Multicellularity Sequencing Project	CAOG_07807
Chaetomorpha coliformis	GenBank	ACL97368.1
Chara australis	GenBank	ABQ81936.1
Coccomyxa subellipsoidea	DOE Joint Genome Institute	jgi|Chlvu1|35848|estExt_Genewise1.C_30373
Colpoda inflata	GenBank	AAD03253.1
Corallomyxa tenera	GenBank	ADK90072.1
Cryptococcus neoformans	GenBank	XM_767104.1
Cryptosporidium parvum	GenBank	XM_001388307.1
Cyanidioschyzon merolae	GenBank	BAC67667.1
Cyanophora paradoxa	GenBank	AF092951.1
Dictyostelium discoideum	GenBank	XP_645839.1
Dimastigella trypaniformis	GenBank	ACO50124.1
Drosophila melanogaster	GenBank	NP_477375.1
Ectocarpus siliculosus	GenBank	CBJ32893.1
Entosiphon sulcatum	GenBank	ACO50125.1
Euglena gracilis	GenBank	ACO50110.1
Galdieria sulphuraria	Michigan State University Galdieria Database	GS01080
Giardia lamblia	GenBank	XP_001704529.1
Gregarina niphandrodes	GenBank	AFNH01001746.1
Guillardia theta	DOE Joint Genome Institute	jgi|Guith1|84077|estExt_Genewise1Plus.C_30325
Hexamita inflata	GenBank	U37081.1
Histiona aroides	TBestDB	HAL00000058
Homo sapiens	GenBank	NP_001949.1
Ignatius tetrasporus	GenBank	ACL97363.1
Jakoba bahamiensis	TBestDB	JBL00000183
Leishmania major	GenBank	XP_001682258.1
Malawimonas californiana	TBestDB	MCL00000750
Malawimonas jakobiformis	TBestDB, GenBank	MJL00000647, DQ295227.1
Mallomonas rasilis	GenBank	AAX09601.1
Mastigamoeba balamuthi	TBestDB	MBL00000044
Monosiga ovata	TBestDB	MNL00000153
Naegleria gruberi	DOE Joint Genome Institute	jgi|Naegr1|56672|estExt_fgeneshHS_pg.C_20241
Nannochloropsis gaditana	Nannochloropsis Genome Project	Nga02886
Nematostella vectensis	GenBank	AB126336.1
Neocallimastix patriciarum	GenBank	EH432084.1, EH432103.1, EH432107.1, EH432109.1, EH432097.1, EH432118.1, EH432108.1, EH431334.1, EH432079.1, EH431347.1, EH431566.1, EH432018.1, EH431651.1, EH431466.1, EH431201.1, EH432044.1, EH432035.1, EH432076.1, EH432048.1, EH431583.1, EH431639.1, EH431436.1, EH431602.1
Neoparamoeba branchiphila	GenBank	ACO50132.1
Paramecium tetraurelia	GenBank	XM_001426894.1
Paulinella chromatophora	GenBank	BAJ14650.1
Perkinsiella amoebae	GenBank	ACO50133.1
Phaeodactylum tricornutum	DOE Joint Genome Institute	jgi|Phatr2|13878|e_gw1.13.85.1
Plasmodium falciparum	GenBank	XP_001350281.1
Porphyra yezoensis	GenBank	AB098024.1
Pythium sp. *quercum*	GenBank	ABI83804.1
Reclinomonas americana	TBestDB	RAL00001686
Rhizopus oryzae	Broad Institute, Rhizopus oryzae Database	RO3G_08488
Rhynchobodo ATCC50359	GenBank	ACO50135.1

Rhynchomonas nasuta	GenBank	ACO50121.1
Saccharomyces cerevisiae	GenBank	NP_009676.1
Sawyeria marylandensis	TBestDB	SML00000003
Spirogyra sp. FWAC125	GenBank	ABQ81941.1, ABQ81942.1
Spironucleus muris	GenBank	U94405.1
Stentor coeruleus	GenBank	AAD03262.1
stramenopile AAU94657.1	GenBank	AAU94657.1
Telonema sp. YPF702	GenBank	BAL63503.1
Tetrahymena thermophila	GenBank	XM_001032213.2
Theileria annulata	GenBank	NW_001091929.1
Toxoplasma gondii	GenBank	XP_002369268.1
Trichomonas vaginalis	GenBank	XP_001325483.1
Trichoplax adhaerens	DOE Joint Genome Institute	jgi|Triad1|36635|estExt_Genewise1.C_210042
Trimastix pyriformis	TBestDB	TPL00002905
Trypanosoma cruzi	GenBank	XP_804709.1
Vitrella brassicaformis	Marine Microbial Eukaryote Transcriptome Sequencing Project	MMETSP0288-20130426|9258

EFL		
Name	Database	Accession
Acanthoeca spectabilis	GenBank	ACD74791.1
Achnanthes kuwaitensis	GenBank	BAG30802.1
Alexandrium ostenfeldii	GenBank	HO658306.1, HO663348.1, HO659275.1, HO663080.1, HO663249.1, HO660709.1, HO654698.1, HO658436.1, HO657895.1, HO658699.1, HO659289.1, HO653990.1, HO660459.1, HO659554.1, HO653259.1, HO663458.1, HO655795.1, HO662048.1, HO652731.1, HO659833.1, HO661052.1, HO661042.1, HO663148.1, HO660391.1, HO661900.1, HO657998.1, HO655571.1, HO661530.1, HO660939.1
Amphidinium carterae	GenBank	CF064659.1
Basidiobolus ranarum	GenBank	ABB84535.1
Bigelowiella natans	GenBank	EU810324.1
Bigelowiella natans 1	DOE Joint Genome Institute	jgi|Bigna1|48294|estExt_Genewise1.C_250046
Bigelowiella natans 2	DOE Joint Genome Institute	jgi|Bigna1|36951|e_gw1.17.41.1
Blastocladiella emersonii	GenBank	EF064246.1
Chaetoceros didymus	GenBank	BAG30804.1
Chlamydomonas reinhardtii	DOE Joint Genome Institute	jgi|Chlre3|132905|estExt_gwp_1H.C_780011
Chlorella variabilis	DOE Joint Genome Institute	jgi|ChlNC64A_1|30705|estExt_Genewise1.C_70295
Chlorococcum NEPCC478	GenBank	EF551323.1
Chondrus crispus	GenBank	CO653259.1, CO649783.1, CO651752.1, CO652990.1, CO651454.1, CO652274.1, CO649837.1, CO650625.1, CO652099.1, CO653172.1, CO652788.1, CO649943.1
Chromera velia	Marine Microbial Eukaryote Transcriptome Sequencing Project	MMETSP0290-20130426|15931
Collozoum amoeboides	GenBank	BAM28641.1
Cryptomonas ovata	GenBank	AB332030.1
Cylindrotheca closterium	GenBank	BAG30805.1
Dictyocoryne profunda	GenBank	BAM28639.1
Diplonema papillatum	TBestDB	DPL00000038
Emiliania huxleyi	GenBank	CV068986.1
Entomophthora muscae	GenBank	ABB84538.1
Eucheuma denticulatum	GenBank	GR427904.1, GR433766.1, GR430211.1, GR430945.1, GR427588.1, GR431549.1, GR433700.1
Eucyrtidium acuminatum	GenBank	BAM28638.1
Fragilariopsis cylindrus	DOE Joint Genome Institute	jgi|Fracy1|269944|estExt_fgenesh2_kg.C_90222
Goniomonas amphinema	GenBank	AB332031.1
Gracilaria changii	GenBank	DV965525.1, DV963172.1, DV963090.1, DV968745.1, DV963156.1, DV966484.1, DV967969.1, DV964985.1, DV965095.1, DV964877.1, DV966975.1, DV969684.1, DV964639.1, DV966046.1
Gromia spaerica	GenBank	SRR071237
Guillardia theta	DOE Joint Genome Institute	jgi|Guith1|149923|fgenesh2_kg.2_#_103_#_1003_1_CCFI_CCFN_EXTA_EXTB
Gymnochlora stellata	GenBank	ACF24595.1
Gymnochlora stellata 1	GenBank	EU810316.1
Gymnochlora stellata 2	GenBank	ACF24585.1
Helicosporidium sp.	GenBank	AY729488.1
Heterocapsa triquetra	GenBank	AY729485.1
Heterophrys marina	GenBank	AFA56270.1
Isochrysis galbana	TBestDB	ISL00000533
Karenia brevis	GenBank	EX876503.1, EX875229.1, EX961328.1, EX961327.1, EX875228.1, EX876502.1, EX972320.1, EX972319.1
Karlodinium micrum	TBestDB	KML00002411

Leucocryptos marina	GenBank	BAJ10903.1
Lingulodinium polyedrum	GenBank	CD809674.1
Lotharella vacuolata	GenBank	ACF24598.1
Lotharella vacuolata 1	GenBank	EU810321.1
Lotharella vacuolata 2	GenBank	ACF24588.1
Mesostigma viride	GenBank	DN254721.1, EC732354.1, EC731514.1, EC731433.1, EC731954.1, EC731134.1, DN254248.1, DN254627.1, DN256785.1, DN256319.1, DN258988.1
Microallomyces sp.	GenBank	ABB84542.1
Microheliella maris	GenBank	AFA56271.1
Micromonas pusilla	DOE Joint Genome Institute	jgi|MicpuC2|33445
Monosiga brevicollis	GenBank	AY026073.1
Neobodo saliens	GenBank	ACO50138.1
Nephroselmis olivacea	GenBank	ACN59934.1
Ochlochaete hystrix	GenBank	ACL97364.1
Olpidium brassicae	GenBank	ABB84544.1
Ostreococcus tauri	DOE Joint Genome Institute	jgi|Ostta4|23014|estExt_fgenesh1_pm.C_Chr_13.00010011
Oxyrrhis marina	TBestDB	OML00001433
Palpitomonas bilix	GenBank	BAL63502.1
Pavlova lutheri	TBestDB	PLL00000038
Perkinsus marinus	GenBank	EER13701.1
Petalomonas cantuscygni	GenBank	ACO50134.2
Phaeocystis antarctica	GenBank	gnl|ti|2192857123, gnl|ti|2192513925
Prototheca wickerhamii	TBestDB	PWL00000297
Prymnesium parvum	GenBank	DV097486.1
Pythium ultimum	Pythium Genome Database	scf1117875581981
Raphidiophrys contractilis	GenBank	AB332032.1
Reticulomyxa filosa	GenBank	ACF24599.1
Rhodomonas salina	GenBank	DQ659244.1
Rhynchopus euleeides	GenBank	ACO50120.1
Rozella allomycis	GenBank	ABB84537.1
Salpingoeca pyxidium	GenBank	ACD74798.1
Salpingoeca rosetta	Broad Institute, Origins of Multicellularity Sequencing Project	PTSG_02256
Scenedesmus obliquus	TBestDB	SOL00000077
Scherffelia dubia	GenBank	AJ919303.1
Sphaeroforma arctica	Broad Institute, Origins of Multicellularity Sequencing Project	SARC_12681
Spizellomyces punctatus	Broad Institute, Origins of Multicellularity Sequencing Project	SPPG_08407
Tetraselmis tetrathele	GenBank	EF551330.1
Thalassionema nitzschioides	GenBank	BAG30809.1
Thalassiosira pseudonana	DOE Joint Genome Institute	jgi|Thaps3|41829|estExt_gwp_gw1.C_chr_100079
Thaumatomastix sp.	GenBank	BAJ14652.1
Trypanoplasma borreli	GenBank	ACO50139.1
Ulva intestinalis	GenBank	EF551324.1
uncultured eukaryote EU810323.1	GenBank	EU810323.1
Volkanus costatus	GenBank	ACD74794.1

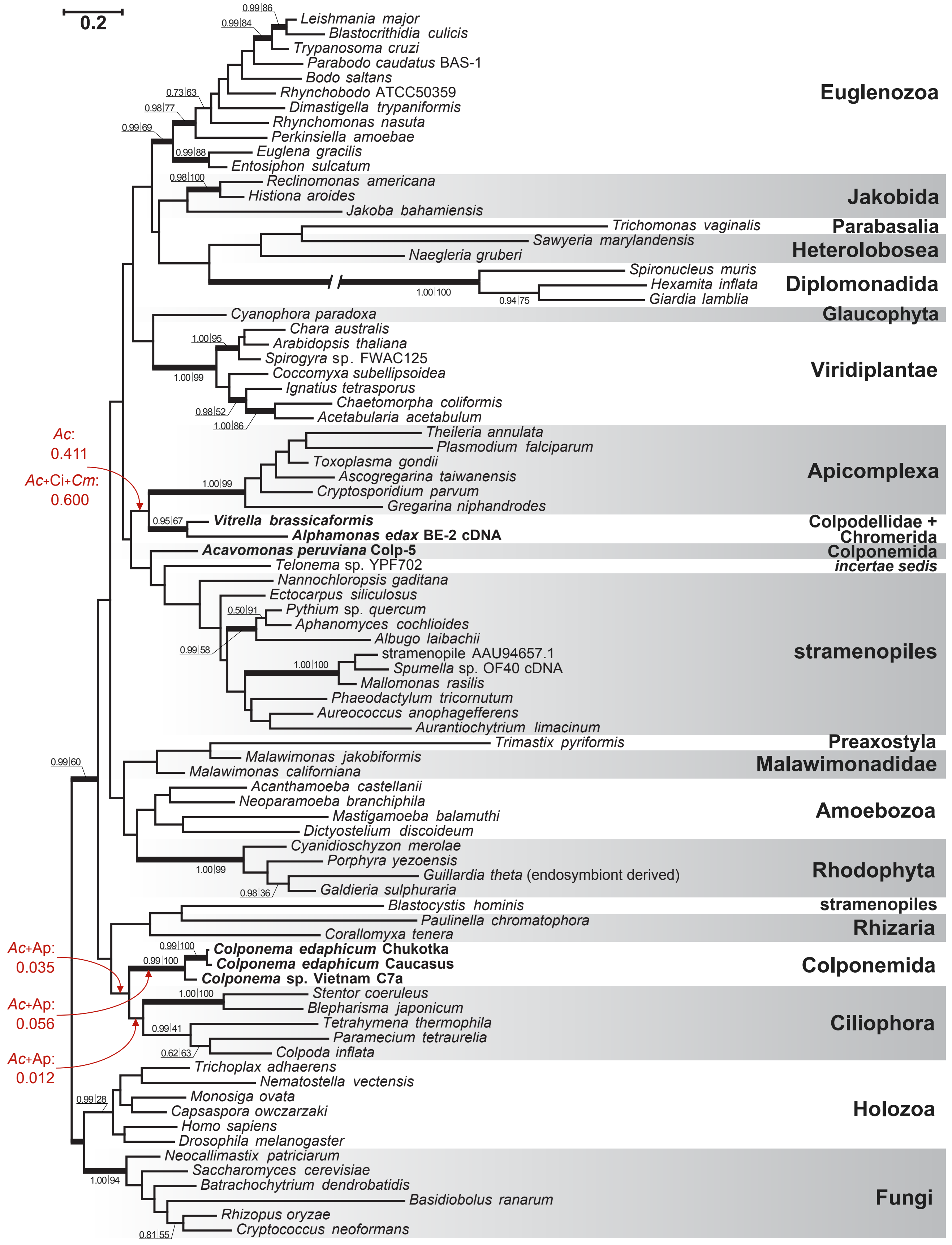

0.2
0.99|86
0.99|84
Leishmania major
Blastocrithidia culicis
Trypanosoma cruzi
Parabodo caudatus BAS-1
Bodo saltans
Rhynchobodo ATCC50359
0.73|63
0.98|77
Dimastigella trypaniformis
Rhynchomonas nasuta
0.99|69
Perkinsiella amoebae
0.99|88
Euglena gracilis
Entosiphon sulcatum
Euglenozoa
0.98|100
Reclinomonas americana
Histiona aroides
Jakoba bahamiensis
Jakobida
Trichomonas vaginalis
Parabasalia
Sawyeria marylandensis
Naegleria gruberi
Heterolobosea
Spironucleus muris
Hexamita inflata
Giardia lamblia
1.00|100
0.94|75
Diplomonadida
Cyanophora paradoxa
Glaucophyta
Chara australis
1.00|95
Arabidopsis thaliana
Spirogyra sp. FWAC125
1.00|99
Coccomyxa subellipsoidea
Ignatius tetrasporus
Chaetomorpha coliformis
0.98|52
Acetabularia acetabulum
1.00|86
Viridiplantae
Ac: 0.411
Ac+Ci+Cm: 0.600
Theileria annulata
Plasmodium falciparum
Toxoplasma gondii
Ascogregarina taiwanensis
1.00|99
Cryptosporidium parvum
Gregarina niphandrodes
Apicomplexa
0.95|67
Vitrella brassicaformis
Alphamonas edax BE-2 cDNA
Colpodellidae + Chromerida
Acavomonas peruviana Colp-5
Colponemida
Telonema sp. YPF702
incertae sedis
Nannochloropsis gaditana
Ectocarpus siliculosus
0.50|91
Pythium sp. quercum
Aphanomyces cochlioides
Albugo laibachii
0.99|58
stramenopile AAU94657.1
1.00|100
Spumella sp. OF40 cDNA
Mallomonas rasilis
Phaeodactylum tricornutum
Aureococcus anophagefferens
Aurantiochytrium limacinum
stramenopiles
Trimastix pyriformis
Preaxostyla
0.99|60
Malawimonas jakobiformis
Malawimonas californiana
Malawimonadidae
Acanthamoeba castellanii
Neoparamoeba branchiphila
Mastigamoeba balamuthi
Dictyostelium discoideum
Amoebozoa
Cyanidioschyzon merolae
Porphyra yezoensis
1.00|99
Guillardia theta (endosymbiont derived)
0.98|36
Galdieria sulphuraria
Rhodophyta
Blastocystis hominis
stramenopiles
Paulinella chromatophora
Corallomyxa tenera
Rhizaria
Ac+Ap: 0.035
0.99|100
Colponema edaphicum Chukotka
0.99|100
Colponema edaphicum Caucasus
Colponema sp. Vietnam C7a
Colponemida
Ac+Ap: 0.056
1.00|100
Stentor coeruleus
Blepharisma japonicum
Tetrahymena thermophila
0.99|41
Paramecium tetraurelia
0.62|63
Colpoda inflata
Ciliophora
Ac+Ap: 0.012
Trichoplax adhaerens
Nematostella vectensis
0.99|28
Monosiga ovata
Capsaspora owczarzaki
Homo sapiens
Drosophila melanogaster
Holozoa
Neocallimastix patriciarum
Saccharomyces cerevisiae
1.00|94
Batrachochytrium dendrobatidis
Basidiobolus ranarum
Rhizopus oryzae
Cryptococcus neoformans
0.81|55
Fungi

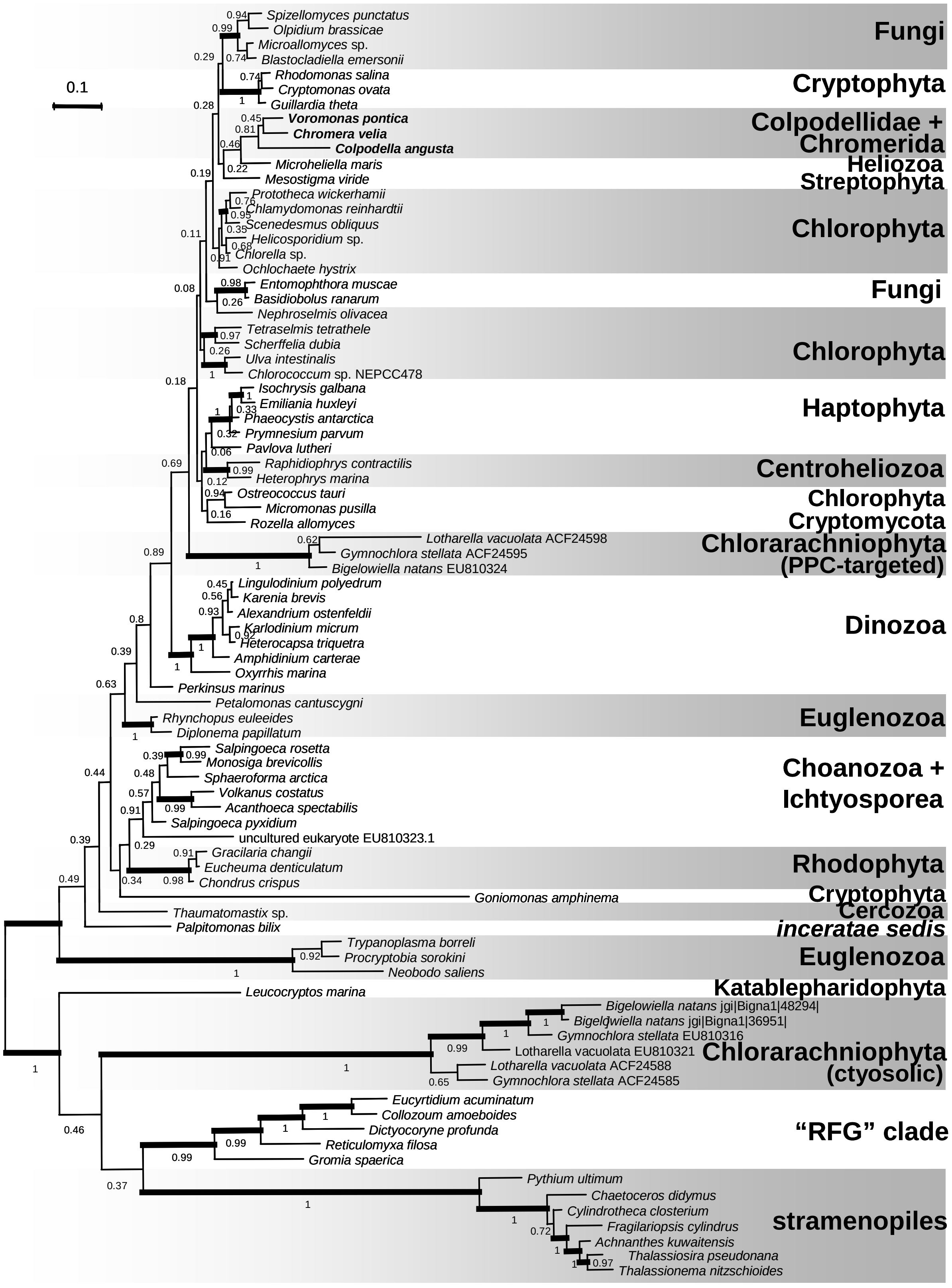

Supplementary Figure S2

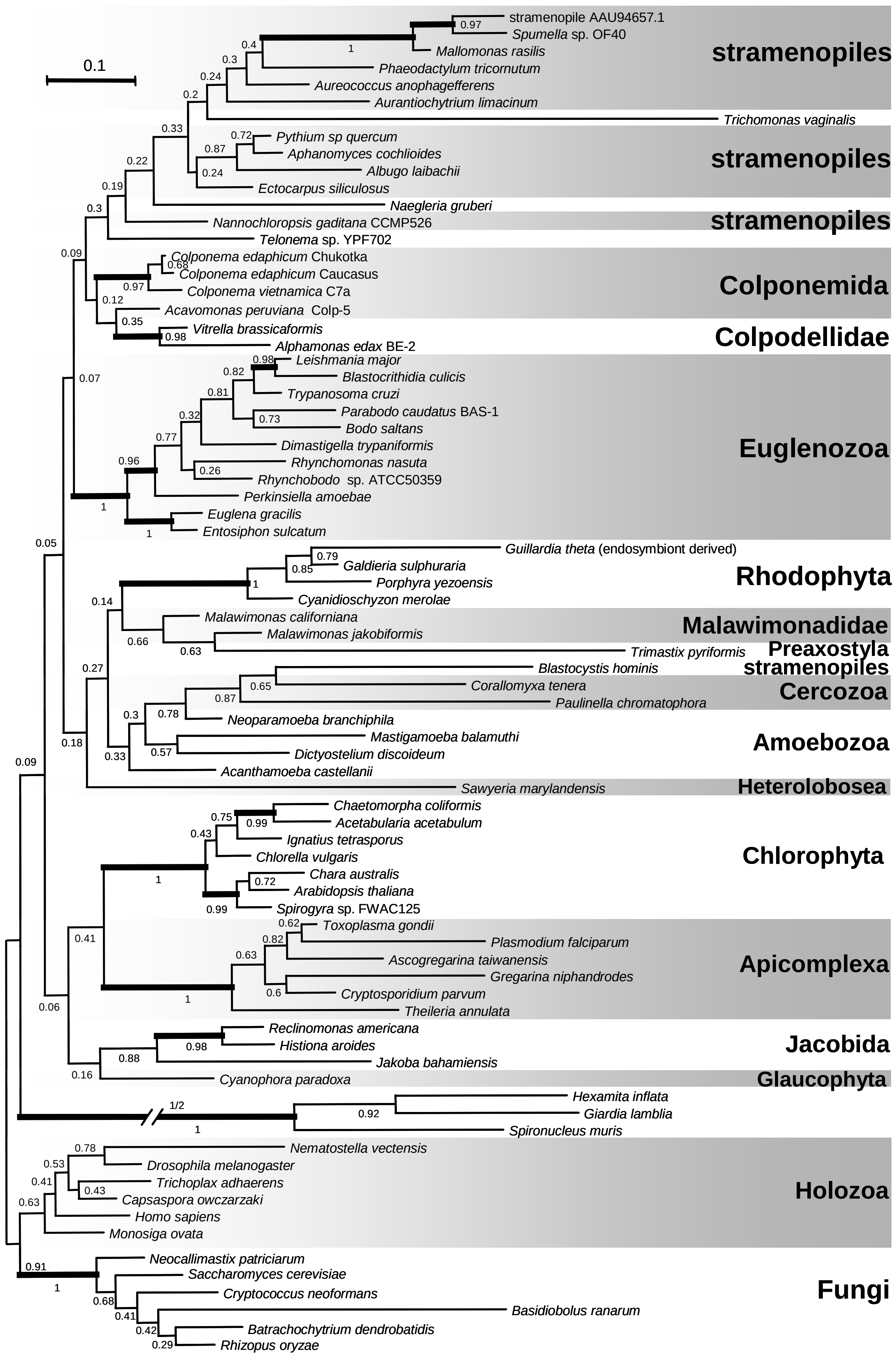

Supplementary Figure S3

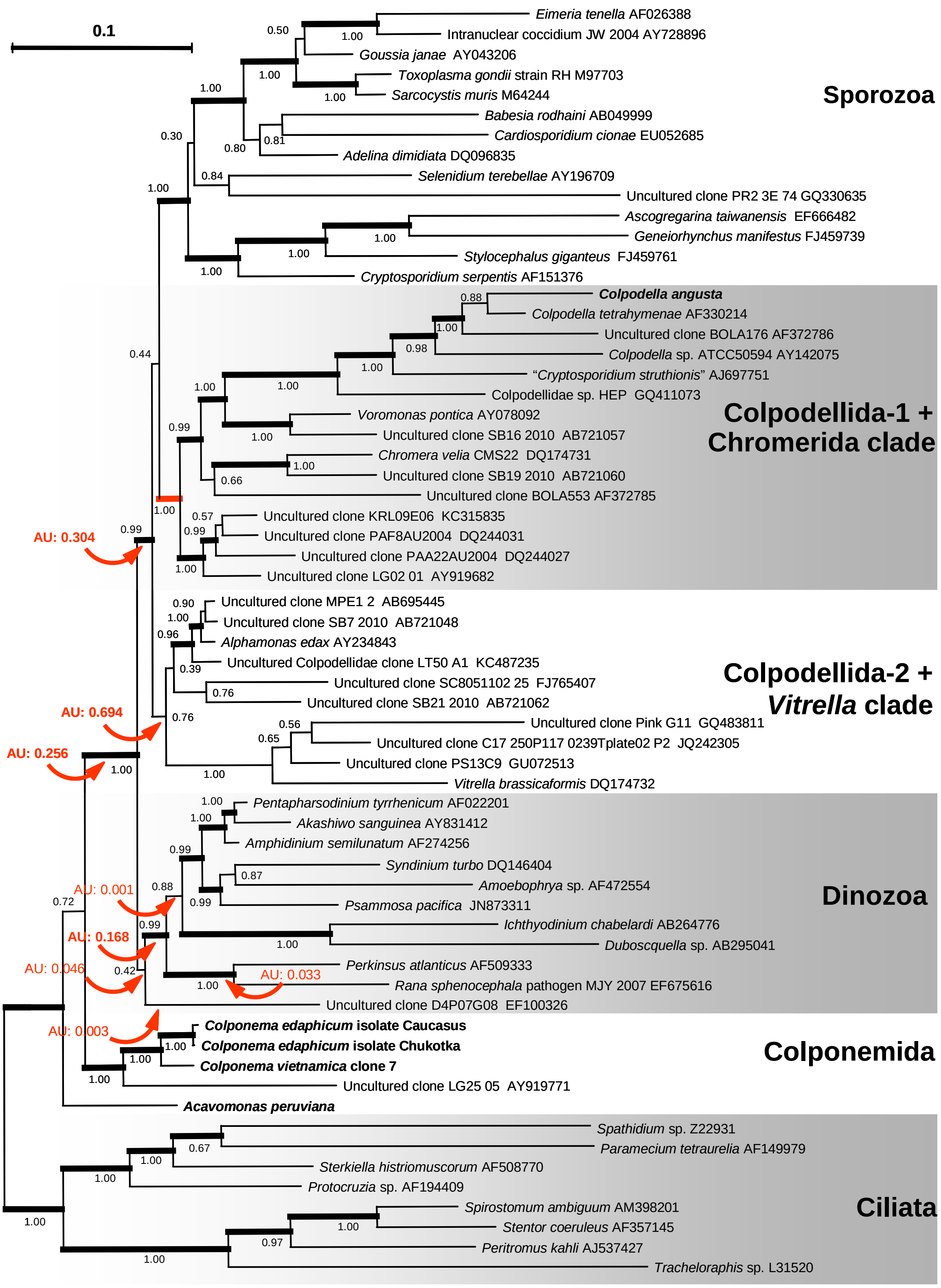

Supplementary Figure S4
